# Cross-cultural asymmetries in oculomotor interference elicited by gaze distractors belonging to Asian and White faces

**DOI:** 10.1038/s41598-021-99954-x

**Published:** 2021-10-14

**Authors:** Xinyuan Zhang, Mario Dalmaso, Luigi Castelli, Shimin Fu, Giovanni Galfano

**Affiliations:** 1 Department of Developmental and Social Psychology, University of Padova, 35131 Padua, Italy; 2 Department of Psychology and Center for Brain and Cognitive Sciences, School of Education, Guangzhou University, Guangzhou, 510006 China

**Keywords:** Psychology, Human behaviour

## Abstract

The averted gaze of others triggers reflexive attentional orienting in the corresponding direction. This phenomenon can be modulated by many social factors. Here, we used an eye-tracking technique to investigate the role of ethnic membership in a cross-cultural oculomotor interference study. Chinese and Italian participants were required to perform a saccade whose direction might be either congruent or incongruent with the averted-gaze of task-irrelevant faces belonging to Asian and White individuals. The results showed that, for Chinese participants, White faces elicited a larger oculomotor interference than Asian faces. By contrast, Italian participants exhibited a similar oculomotor interference effect for both Asian and White faces. Hence, Chinese participants found it more difficult to suppress eye-gaze processing of White rather than Asian faces. The findings provide converging evidence that social attention can be modulated by social factors characterizing both the face stimulus and the participants. The data are discussed with reference to possible cross-cultural differences in perceived social status.

## Introduction

The averted gaze of another individual is known to provide humans critical information about the environment, such as the presence of potential threats or rewards, and prompts them to react appropriately^1,2^. The ability to process eye gaze can also have a crucial role to help deciphering the intentions and mental states of others^3,4^. Consistent with this view, a wealth of data has suggested that gaze deviations of others trigger a reflexive orienting of attention in the same direction^5–7^. This finding has been consistently reported using the so-called gaze-cueing paradigm, in which a face with direct gaze suddenly shifts the eyes either leftwards or rightwards and is followed by a target stimulus randomly appearing either at the gazed-at location or at the opposite location. Irrespective of the specific task, manual performance is typically better when the target appears at the gazed-at location (spatially-congruent trials) than at the nongazed-at location (spatially-incongruent trials), likely as a consequence of the fact that the averted gaze caused a shift of attention in the corresponding direction^6^. In recent years, evidence has accumulated suggesting that the pushing of attention exerted by an averted gaze is not entirely automatic, in that it is sensitive to different social variables characterizing both the face stimulus, the participants, and their relationship^8^. For instance, a larger gaze-cueing effect has been reported for faces more similar to those of the participants^9,10^, for familiar over unfamiliar faces^11^, for the faces of politicians of one’s own political affiliation^12^, as well as for faces depicting either dominant^13^ or high-status individuals^14,15^.

In general, the effects of social factors call into play a complex interaction between variables related to both the face stimulus and the participants^16,17^. This is particularly evident in the context of ethnic membership, in which basic intergroup dynamics can interact with other social variables such as perceived social status. In this regard, Pavan et al.^18^ tested White and Black participants in a gaze-cueing paradigm which included faces of both White and Black individuals. The results showed that Black participants exhibited a similar gaze-cueing effect in response to both White and Black faces, whereas White participants displayed no gaze-cueing effect when exposed to Black faces. This pattern has been later replicated by Weisbuch et al.^19^, who also provided direct evidence that this asymmetry was likely to reflect well-known differences in the social status associated with different ethnic groups, as shown in previous studies^20^. In this regard, it is worth noting that ethnicity-based status differences may change as a function of the social context in which they are assessed. Indeed, there is evidence that, in China, White individuals are associated with a higher social status as compared to Chinese individuals^21,22^. On the other hand, in a sample of Italian respondents, no significant differences in perceived status were observed while judging White and Chinese people^22^.

Additional evidence supporting the view that the influence of ethnicity on gaze cueing does not follow a simple ingroup-bias dynamic comes from a recent study conducted by Zhang et al.^22^, in which Italian and Chinese participants were tested in a manual response task, with faces depicting both White and Asian individuals (i.e., faces that could be unambiguously perceived as either Italian or Chinese). In the case of the Italian sample, a reliable gaze-cueing effect was observed irrespective of face ethnicity, and its magnitude was similar for both White and Asian faces. Interestingly, in the case of Chinese participants, the results showed an outgroup-like bias, in that a reliable gaze-cueing effect emerged for White faces, whereas the data suggested the presence of an overall null gaze-cueing effect for Asian faces. This latter result was unexpected in that, in so far, no study addressing the specific effect of ethnicity on gaze cueing has reported such a pattern for faces belonging to one’s ingroup^8,18,19^.

The aim of the present study was to investigate the peculiar pattern of findings reported by Zhang et al.^22^ in more detail, using richer and more sensitive measures of spatial attention, i.e., eye movements, which can provide novel insights underlying eye-gaze processing^23,24^. Importantly, evidence has accumulated indicating that saccadic selection represents a more sensitive measure than manual selection in several visual tasks which directly involve attentional control^25^, including spatial cueing paradigms^26,27^. In recent years, it has been shown that the gaze-cueing effect can be effectively captured by means of experimental paradigms that focus on eye movement dynamics, i.e., more direct, online measures of attentional orienting with respect to manual responses^28–31^.

Thus, in the present study, we relied on eye movements measures of social attention and adopted the oculomotor interference paradigm developed by Ricciardelli et al.^29^, which has proved to be both robust^32–34^, and well-suited to uncover social modulations of the gaze-cueing effect^16,35,36^. In this paradigm, individuals are asked to perform a saccadic eye movement either to the left or to the right according to an instruction cue provided at fixation on every trial. A task-irrelevant central face is presented displaying an averted gaze either looking to the same (spatially-congruent trials) or the opposite location (spatially-incongruent trials) as the instructed saccadic direction. Given that spatially congruent and incongruent trials occur with the same probability, eye gaze direction of the central face represents a distractor stimulus that participants are explicitly instructed to ignore. Notwithstanding, the results typically show that performance is better (in terms of both saccadic latency and accuracy) for spatially-congruent trials than for spatially-incongruent trials. This suggests that, when the instructed saccade has the same spatial vector as the averted gaze, the task is easier with respect to when the instructed saccade and the averted gaze have opposite spatial vectors. Thus, participants seem to be unable to ignore the direction of the task-irrelevant gaze stimulus, which, in turn, gives rise to oculomotor interference^29,33^. Interestingly, this paradigm also enables to conduct complementary analyses that provide hints about both the voluntary and reflexive components underlying saccadic programming^33–35^.

Here, the oculomotor interference paradigm was used with faces belonging to different ethnicities, i.e., White and Asian. The first goal of Experiment 1 was to further investigate the hypothesis that the gaze-cueing effect is stronger in response to White vs. Asian faces in a Chinese sample. As a second major goal, we aimed to more deeply explore whether Chinese participants do not exhibit a gaze-cueing effect when presented with Asian faces (i.e., ingroup faces). Here, using eye-tracking techniques and focusing on eye movement measures, we expected to find evidence in favour of the idea that also ingroup faces can actually orient spatial attention in a sample of Chinese participants, as evidenced by a significant oculomotor interference effect. In other words, Chinese respondents were expected to exhibit faster saccadic responses on spatially-congruent trials than on spatially-incongruent trials also for ingroup faces.

**Figure 1:**
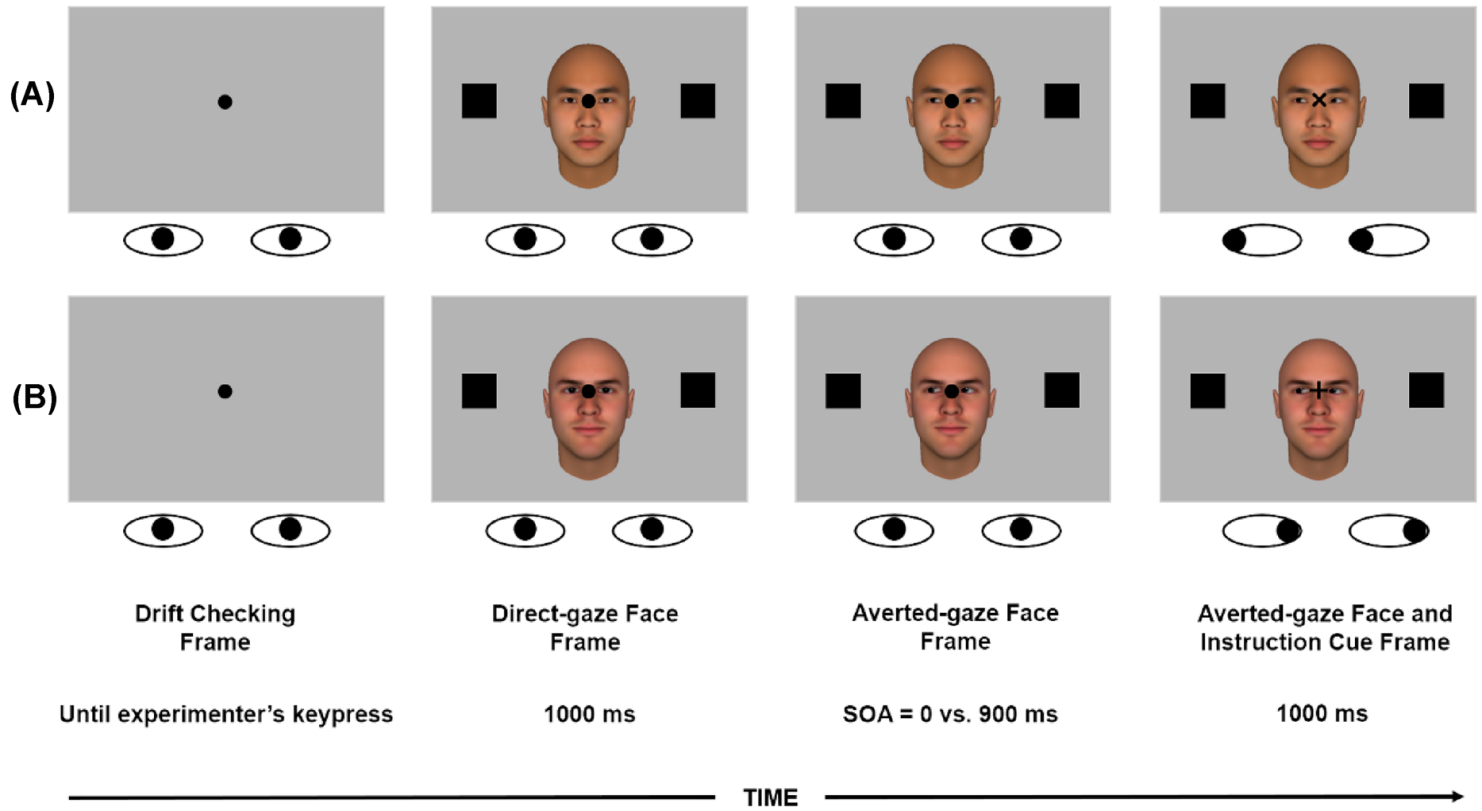
Trial sequence and examples of stimuli: (**A**) An Asian face distractor in a spatially-congruent trial where the “×” symbol indicates a leftward saccade and the central gaze is averted to the left; (**B**) A White face distractor in a spatially-incongruent trial where the “+” symbol indicates a rightward saccade and the central gaze is averted to the left. Stimuli are not drawn to scale. Schematic eyes depict the correct gaze behavior that participants were instructed to execute. The figure was created with Gimp 2.10 (https://www.gimp.org/).

In addition, in keeping with previous studies^29,34^, we manipulated the Stimulus Onset Asynchrony (SOA) between the onset of the averted gaze and the onset of the instruction cue. At the short SOA, the instruction cue was synchronous to the onset of the averted gaze (i.e., SOA = 0 ms), while at the long SOA, the instruction cue appeared 900 ms after presentation of the averted-gaze face (see Fig. 1). This manipulation was aimed at addressing the time-course of oculomotor interference and its possible modulations as a function of the ethnicity of the faces. In this regard, Dalmaso et al.^37^, who were interested in comparing White and Black faces with White participants, have shown that the gaze of White faces elicits a larger oculomotor interference than that belonging to Black faces, but this difference was only detectable with a short SOA. This is likely to reflect that social information, despite being task-irrelevant, is initially always extracted from faces but, at the long SOA, it tends to be no longer active due to either spontaneous decay or endogenous suppression. To sum up, we expected Asian participants to exhibit an oculomotor interference in response to both White and Asian faces, even though, based on the results reported by Zhang et al.^22^, the effect might turn up to be stronger in the former case, likely as a consequence of differences in the perceived social status associated to the two groups. We predicted that this pattern might be specifically present at the short SOA, because at the long SOA social information extracted from the face may be subjected to decay, being irrelevant for the task at hand. A further goal of the present paper, addressed in Experiment 2, was to assess whether, in a sample of Italian individuals, oculomotor interference is present irrespective of the White vs. Asian category membership of the faces, in line with previous studies using manual response paradigms^22,38^. In other words, the magnitude of oculomotor interference was expected to be similar regardless of the ethnicity of the face used as stimulus.

## Results

In order to test the key hypotheses laid down in the introduction, data from the two experiments were first analysed separately. In a second step, given that the experiments relied on the same procedure, a combined analysis was also conducted.

## Chinese participants

### Saccadic latencies

**Figure 2:**
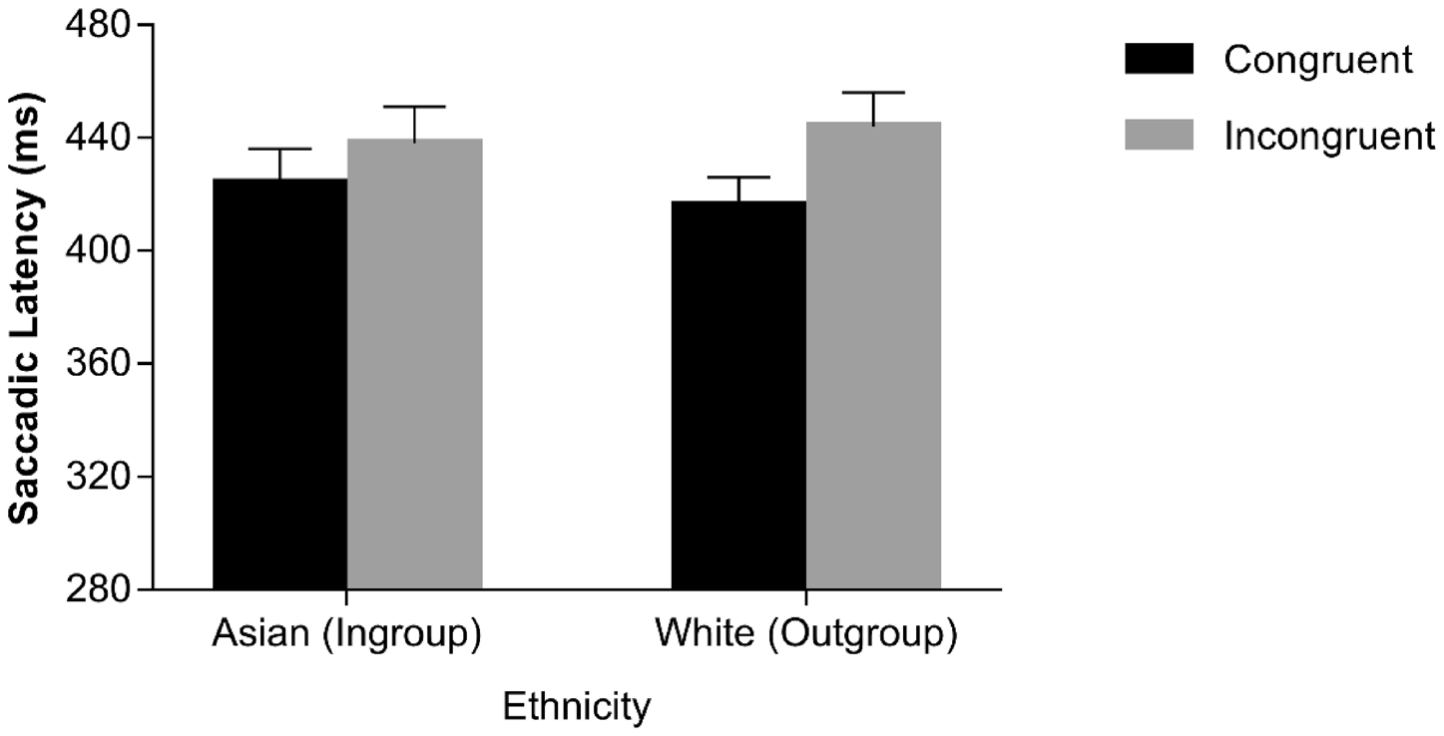
Median saccadic latencies as a function of spatial congruency and face ethnicity in the sample of Chinese participants at the 0-ms SOA. Error bars represent Standard Errors.

A 2 × 2 × 2 repeated-measures ANOVA with congruency (spatially congruent vs. incongruent), ethnicity (Asian vs. White), and SOA (0 vs. 900 ms) as within-participant factors was conducted on median saccadic latencies for correct responses^33^. The main effect of congruency was significant, *F*(1, 29) = 10.078, *p* = 0.004, *η*^2^_*p*_ = 0.258, with shorter latencies on congruent trials (*M* = 402 ms, *SE* = 11.75) than on incongruent trials (*M* = 411 ms, *SE* = 12.13). SOA also yielded a significant main effect, *F*(1, 29) = 151.598, *p* < 0.001, *η*^2^_*p*_ = 0.839, with shorter latencies at the 900-ms SOA (*M* = 383 ms, *SE* = 12.79) than at the 0-ms SOA (*M* = 430 ms, *SE* = 11.20). The congruency × SOA interaction was significant, *F*(1, 29) = 12.309, *p* = 0.001, *η*^2^_*p*_ = 0.298, indicating that the difference between congruent and incongruent trials was greater at the 0-ms SOA, *t*(29) = 4.940, *p* < 0.001, *d* = 1.017, than at the 900-ms SOA, *t*(29) = 0.877, *p* = 0.388, *d* = 0.198. Importantly, the congruency × SOA × ethnicity interaction was also significant, *F*(1, 29) = 5.631, *p* = 0.024, *η*^2^_*p*_ = 0.163. No other significant results emerged (*F*s < 1.345, *p*s > 0.256). The three-way interaction was further explored through two additional ANOVAs, one for each level of SOA, with congruency and ethnicity as within-participants factors. As for the 0-ms SOA, the main effect of congruency was significant, *F*(1, 29) = 24.399, *p* < 0.001, *η*^2^_*p*_ = 0.457, with shorter latencies on congruent trials (*M* = 420 ms, *SE* = 10.78) than on incongruent trials (*M* = 441 ms, *SE* = 12.01), while the main effect of ethnicity was non-significant, *F*(1, 29) =  0.035, *p* = 0.853, *η*^2^_*p*_ = 0.001. Importantly, the congruency × ethnicity interaction was significant, *F*(1, 29) = 5.392, *p* = 0.027, *η*^2^_*p*_ = 0.157, since the difference between congruent vs. incongruent trials was significant for both White, *t*(29) = 5.937, *p* < 0.001, *d* = 1.211, and Asian faces, *t*(29) = 2.401, *p* = 0.023, *d* = 0.593, but the difference was larger in the former case (29 ms vs. 14 ms). As for the 900-ms SOA, no significant results emerged (*F*s < 0.770, *p*s > 0.387; see also Fig. 2; Table 1).

**Table 1:** Median saccadic latencies (sRT) in milliseconds for correct responses, and percentage of errors (%E), for all experimental cells in Chinese and Italian participants.

	0-ms SOA				900-ms SOA			
	White faces		Asian faces		White faces		Asian faces	
	C	I	C	I	C	I	C	I
**Chinese participants**								
sRT	416 (10)	444 (12)	424 (12)	438 (13)	386 (13)	380 (12)	383 (14)	382 (13)
%E	4.51 (0.99)	14.74 (2.14)	4.24 (0.10)	14.89 (2.40)	7.14 (1.28)	6.45 (1.25)	6.26 (1.29)	7.29 (1.28)
**Italian participants**								
sRT	367 (11)	379 (10)	355 (10)	375 (10)	329 (10)	333 (9)	325 (8)	330 (9)
%E	3.70 (0.80)	10.38 (1.41)	3.14 (0.74)	9.55 (1.32)	4.56 (1.10)	6.11 (1.06)	5.15 (0.83)	6.83 (1.46)

Standard errors are in brackets. C = congruent trials; I = incongruent trials.

### Saccadic directional errors

An ANOVA with the same factors as that conducted on saccadic latencies was also run on errors. Congruency yielded a significant main effect, *F*(1, 29) = 20.313, *p* < 0.001, *η*^2^_*p*_ = 0.412, with fewer errors on congruent trials (*M* = 5.54%, *SE* = 0.90) than on incongruent trials (*M* = 10.84%, *SE* = 1.54). SOA was also significant, *F*(1, 29) = 11.580, *p* = 0.002, *η*^2^_*p*_ = 0.285, with fewer errors at the 900-ms SOA (*M* = 6.79%, *SE* = 1.04) than at the 0-ms SOA (*M* = 9.59%, *SE* = 1.33). The congruency × SOA interaction was significant, *F*(1, 29) = 29.212, *p* < 0.001, *η*^2^_*p*_ = 0.502, indicating that the difference in accuracy between congruent vs. incongruent trials was greater at 0-ms SOA, *t*(29) = 5.286, *p* < 0.001, *d* = 0.965, than at 900-ms SOA, *t*(29) = 0.209, *p* = 0.836, *d* = 0.038. No other significant results emerged (*F*s < 1, *p*s > 0.392), including the theoretically-relevant congruency × SOA × ethnicity interaction (*F* < 1, *p* = 0.599; see also Table 1).

### Reflexive nature of saccades

**Figure 3:**
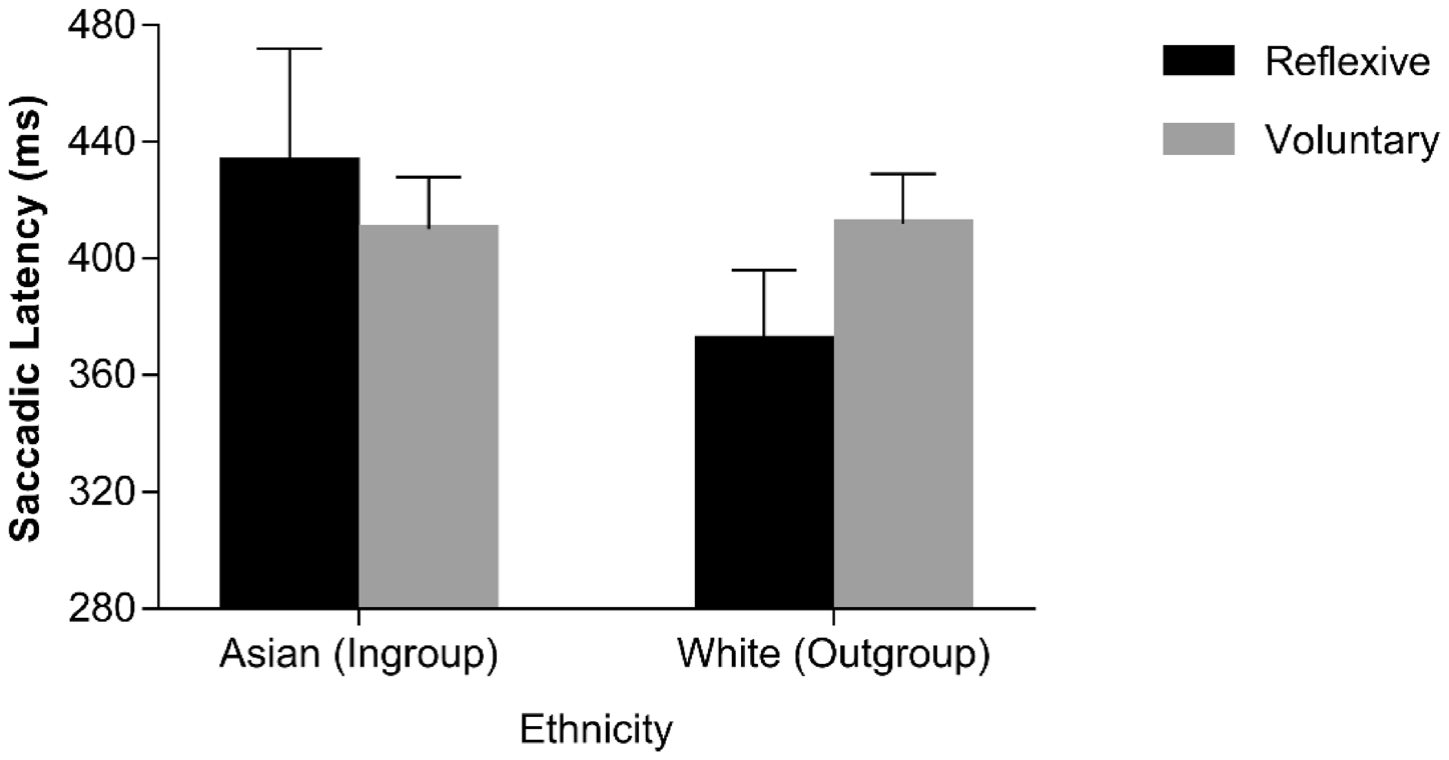
Median saccadic latencies as a function of saccadic type and face ethnicity in the sample of Chinese participants. Error bars represent Standard Errors.

Previous studies^33,35^ exploring gaze following behaviour in the oculomotor interference paradigm have shown that saccades performed on spatially incongruent trials have shorter latencies when they are executed erroneously, i.e., following the direction conveyed by the averted-gaze stimulus (i.e., reflexive saccades) as compared to when they are executed correctly, i.e. following the direction conveyed by the instruction cue (i.e., voluntary saccades). A similar pattern of results was expected also in the present context. Moreover, we also explored whether reflexive vs. voluntary saccades were further shaped by the ethnicity of the distracting face. Only 18 participants showed both voluntary and reflexive saccades in all of the experimental conditions. Because analyses were based on only a subsample of participants, conclusions should be taken with caution. An ANOVA with saccade type (reflexive vs. voluntary), SOA (0 vs. 900 ms) and ethnicity (Asian vs. White) was conducted. As for the main effects, only ethnicity approached the conventional level of statistical significance, *F*(1, 17) = 3.747, *p* = .0.070, *η*^2^_*p*_ = 0.181, reflecting a tendency towards shorter latencies for White faces (*M* = 392 ms, *SE* = 16.51) than for Asian faces (*M* = 421 ms, *SE* = 21.02). Importantly, the saccade type × ethnicity interaction was significant, *F*(1, 17) = 5.590, *p* = 0.030, *η*^2^_*p*_ = 0.247, as latencies of reflexive saccades were shorter than latencies of voluntary saccades for White faces, *t*(17) = 3.110, *p* = 0.006, *d* = 0.603, but not for Asian faces, *t*(17) = − 0.901, *p* = 0.380, *d* = − 0.263 (see Fig. 3). No other sources of variance were significant, (*F*s < 3.038, *p*s > 0.099), including the theoretically-relevant three-way interaction (*F* < 1, *p* = 0.370; see also Fig. 3; Table 2).

**Table 2:** Median saccadic latencies (sRT), in milliseconds, for reflexive (re) and voluntary (vo) saccades in all experimental cells in Chinese and Italian participants.

	0-ms SOA				900-ms SOA			
	White faces		Asian faces		White faces		Asian faces	
	re	vo	re	vo	re	vo	re	vo
**Chinese participants**								
sRT	393 (23)	444 (15)	422 (24)	438 (17)	346 (24)	380 (16)	446 (51)	382 (17)
**Italian participants**								
sRT	358 (22)	379 (13)	376 (27)	375 (12)	301 (12)	333 (11)	313 (17)	330 (12)

Standard errors are in brackets.

## Italian participants

### Saccadic latencies

**Figure 4:**
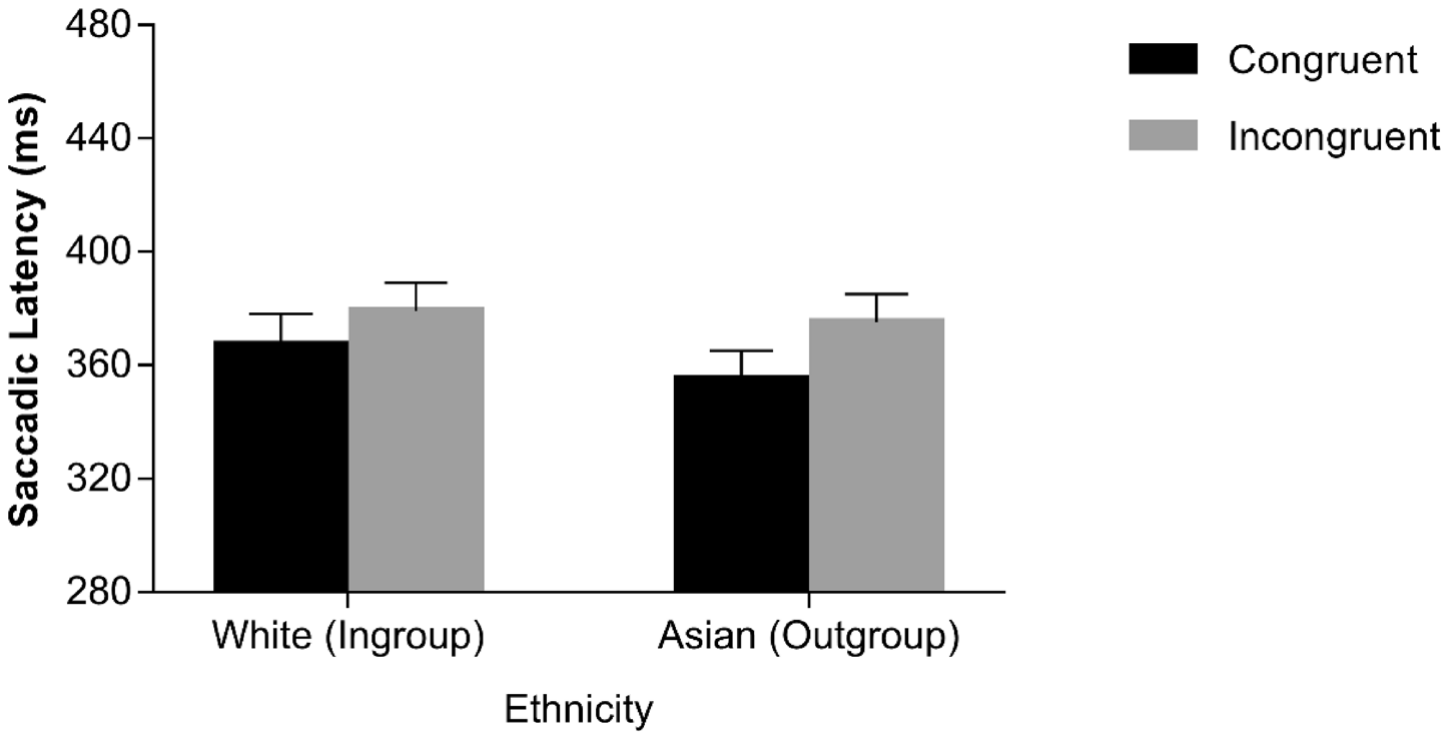
Median saccadic latencies as a function of spatial congruency and face ethnicity in the sample of Italian participants at the 0-ms SOA. Error bars represent Standard Errors.

Median saccadic latencies for correct responses were analysed using the same ANOVA design implemented for Chinese participants. Congruency yielded a significant main effect, *F*(1, 29) = 24.453, *p* < 0.001, *η*^2^_*p*_ = 0.457, with shorter latencies on congruent trials (*M* = 344 ms, *SE* = 9.56) than on incongruent trials (*M* = 354 ms, *SE* = 9.03). The main effect of SOA was also significant, *F*(1, 29) = 115.511, *p* < 0.001, *η*^2^_*p*_ = 0.799, with shorter latencies at the 900-ms SOA (*M* = 329 ms, *SE* = 8.75) than at the 0-ms SOA (*M* = 369 ms, *SE* = 10.04). Ethnicity also yielded a significant main effect, *F*(1, 29) = 7.571, *p* = 0.010, *η*^2^_*p*_ = 0.207, with shorter latencies for Asian faces (*M* = 346 ms, *SE* = 8.91) than White faces (*M* = 352 ms, *SE* = 9.68). The congruency × SOA interaction was significant, *F*(1, 29) = 9.687, *p* = 0.004, *η*^2^_*p*_ = 0.250, indicating that the difference between congruent and incongruent trials was greater at the 0-ms SOA, *t*(29) = 5.71, *p* < 0.001, *d* = 0.980, than at the 900-ms SOA, *t*(29) = 1.78, *p* = 0.086, *d* = 0.297. All the other interactions were non-significant (*F*s < 1.836, *p*s > 0.186), including the theoretically-relevant congruency × SOA × ethnicity interaction (*F* < 1, *p* = 0.498). For completeness, two additional ANOVAs, namely one for each level of SOA, with congruency and ethnicity as within-participants factors, were performed. In both ANOVAs, the congruency × ethnicity interaction was not significant (*F*s < 1.99, *p*s > 0.169; see also Fig. 4; Table 1).

### Saccadic directional errors

Data were analyzed through the same ANOVA design used in latencies analyses. Only congruency yielded a significant main effect, *F*(1, 29) = 26.678, *p* < 0.001, *η*^2^_*p*_ = 0.479, with fewer errors on congruent trials (*M* = 4.13%, *SE* = 0.64) than on incongruent trials (*M* = 8.22%, *SE* = 1.01). The congruency × SOA interaction was also significant, *F*(1, 29) = 10.156, *p* = 0.003, *η*^*2*^_*p*_ = 0.259, indicating that the difference between congruent and incongruent trials was greater at 0-ms SOA, *t*(29) = 5.307, *p* < 0.001, *d* = 0.969, than at 900-ms SOA, *t*(29) = 1.684, *p* = 0.103, *d* = 0.307. No other significant results emerged (*F*s < 1.977, *p*s > 0.170), including the theoretically-relevant congruency × SOA × ethnicity interaction (*F* < 1, *p* = 0.837; see also Table 1).

### Reflexive nature of saccades

**Figure 5:**
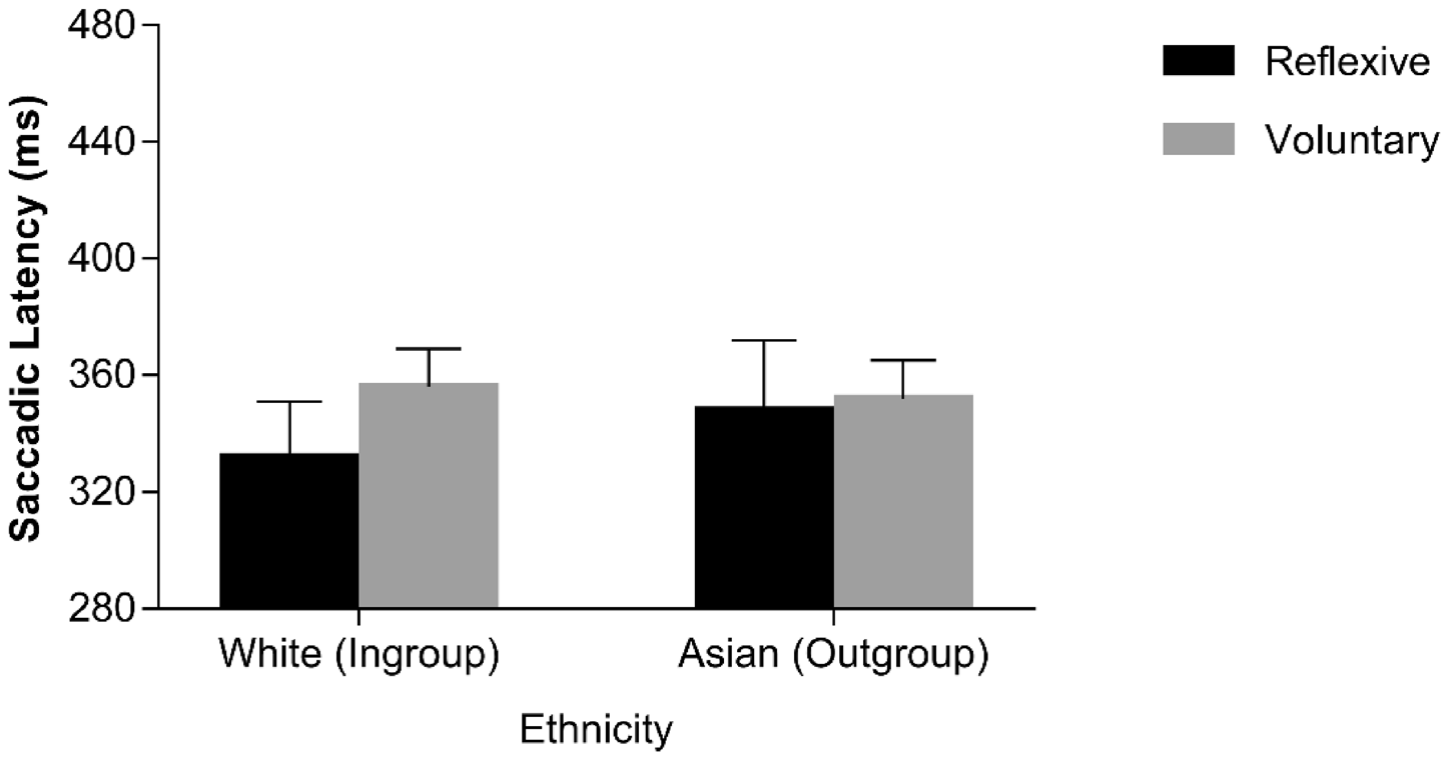
Median saccadic latencies as a function of saccadic type and face ethnicity in the sample of Italian participants. Error bars represent Standard Errors.

Only 18 Italian participants showed both voluntary and reflexive saccades in all of the experimental conditions. Median latencies of reflexive and voluntary saccadic eye movements executed on incongruent trials were therefore analyzed through a repeated-measures ANOVA with saccade type (reflexive vs. voluntary), SOA (0 vs. 900 ms) and ethnicity (White vs. Asian) as within-participant factors. The main effect of saccade type was significant, *F*(1, 17) = 7.496, *p* = 0.014, *η*^2^_*p*_ = 0.306, with shorter latencies associated with reflexive (*M* = 325 ms, *SE* = 17.81) than voluntary (*M* = 344 ms, *SE* = 13.10) saccades. SOA also yielded a significant main effect, *F*(1, 17) = 36.548, *p* < 0.001, *η*^2^_*p*_ = 0.683, with shorter latencies at the 900-ms SOA (*M* = 310 ms, *SE* = 12.33), than at the 0-ms SOA (*M* = 359 ms, *SE* = 18.60). The saccade type × ethnicity interaction approached the conventional level of statistical significance, *F*(1, 17) = 3.146, *p* = 0.094, *η*^2^_*p*_ = 0.156, reflecting the fact that latencies of reflexive saccades tended to be shorter than latencies of voluntary saccades for White faces, *t*(17) = 4.600, *p* < 0.001, *d* = 0.746, but not for Asian faces, *t*(17) = 0.580, *p* = 0.570, *d* = 0.167 (see Fig. 5). All the other results were non-significant, (*F*s < 1.919, *p*s > .184), including the theoretically-relevant three-way interaction (*F* = 1.789, *p* = 0.199; see also Fig. 5; Table 2).

## Comparison between Chinese and Italian participants

For completeness, additional analyses were also performed combining the data from the two experiments, in which the between-participants factor group (Chinese vs. Italian) was added to the ANOVAs addressing saccadic latencies, directional errors, and the reflexive nature of saccades. As for saccadic latencies, the main effects of congruency and SOA, and their interaction, were all significant (*F*s > 20.461, *p*s < 0.001). Moreover, the only significant results involving the group factor were the main effect, *F*(1, 58) = 14.65, *p* < 0.001, *η*^2^_*p*_ = 0.202, indicating that Italians were overall faster than Chinese and, more importantly, the congruency × ethnicity × SOA × group interaction, *F*(1, 58) = 4.711, *p* = 0.034, *η*^2^_*p*_ = 0.075. In order to better understand the latter pattern, two further ANOVAs were conducted separately for the two SOAs. For the 900-ms SOA, the congruency × ethnicity × group interaction was not significant, *F*(1, 58) = 0.163, *p* = 0.688, *η*^2^_*p*_ = 0.003. In contrast, for the 0-ms SOA, the congruency × ethnicity × group interaction yielded a significant effect, *F*(1, 58) = 7.267, *p* = 0.009, *η*^2^_*p*_ = 0.111. Crucially, this latter finding confirmed that at the short SOA, oculomotor interference was modulated by ethnicity in a different way in the two experiments.

As for saccadic directional errors, the main effects of congruency and SOA, and their interaction, were all significant (*F*s > 12.108, *p*s < 0.001). Moreover, the only significant result involving the group factor was the congruency × SOA × group interaction, *F*(1, 58) = 4.748, *p* = 0.033, *η*^2^_*p*_ = 0.076. This reflected a stronger oculomotor interference for Chinese participants at the 0-ms SOA, although this finding was not further qualified by the ethnicity of the faces.

As for the reflexive nature of saccades, the main effects of saccade type, SOA, ethnicity, and the saccade type × ethnicity interaction, were all significant (*F*s > 4.811, *p*s < 0.035). Moreover, the only significant results involving the group factor were the main effect, *F*(1, 34) = 9.81, *p* = 0.004, *η*^2^_*p*_ = 0.224, indicating that Italians were overall faster than Chinese, and the SOA × ethnicity × group interaction, *F*(1, 34) = 4.213, *p* = 0.048, *η*^2^_*p*_ = 0.110. The lack of significance for the saccade type × ethnicity × group interaction, *F*(1, 34) = 1.864, *p* = 0.181, *η*^2^_*p*_ = 0.052, suggests the presence of a similar pattern in the two experiments, consisting of lower latencies for reflexive than voluntary saccades for White faces, but not for Asian faces.

## Discussion

The general goal of the present study was to investigate social attention dynamics in different cultural contexts. By using an oculomotor interference paradigm, we aimed at providing a more refined and robust test of the nature of attentional processing undergone by social cues such as eye gaze of others. According to the picture stemming from studies using covert attention paradigms and manual response tasks, we expected that processing of spatial information conveyed by the gaze of others would result in a different pattern of findings depending on complex interactions related to ethnic membership of both the participant and the face stimulus^18,19,22^. In particular, Zhang and colleagues^22^ have provided evidence that, in Chinese participants, the gaze of ingroup members does not seem to undergo privileged processing leading to enhanced gaze-cueing effects. Indeed, the overall pattern emerging from the data reported by Zhang et al.^22^ seems to suggest a null gaze-cueing effect for Asian faces, despite a significant gaze-cueing effect for White faces. Here, we addressed this issue further by using oculomotor measures, that are known to provide a more stable, robust, and ecologically-valid pattern of spatial attention behaviour with respect to manual responses^25^.

Interestingly, oculomotor measures revealed the presence of a significant gaze-driven attention response in Chinese participants when eye gaze belonged to Asian faces. Moreover, as predicted, Chinese individuals exhibited a greater gaze-following behavior for White than for Asian faces, as testified by a more pronounced oculomotor interference reflected in both saccadic latencies and reflexive saccades. On the contrary, Italian individuals displayed an overall comparable oculomotor interference effect for both White and Asian faces, suggesting a similar gaze-following behavior. These latter findings are in line with those reported by previous studies^22,38^, and extend and confirm this pattern with more direct attentional measures in an oculomotor context. Furthermore, the overall magnitude of oculomotor interference was larger for the 0-ms SOA, i.e., when the instruction cue simultaneously changed with the averted gaze. This result is consistent with previous oculomotor interference studies^29,34^ and is likely to reflect that, with a long SOA, participants have more time to process the instruction cue and disregard the task-irrelevant distractor stimulus. In a related vein, at increasing SOAs, modulations as a function of social variables are expected to decrease, as shown in previous studies^8,13,15,37^, since the activated social knowledge is also task-irrelevant, and hence is likely to either spontaneously decay or to be subjected to top-down suppression. Importantly, these outcomes have been also corroborated by additional analyses directly comparing the two experiments.

In sum, the present findings show that Chinese individuals display an outgroup-like bias in gaze-following behavior so that oculomotor interference is stronger for White than for Asian faces. This pattern is not shown by Italian individuals, who seem to exhibit a similar gaze-driven attention response to White and Asian faces. One possibility is that this asymmetry reflects differences in perceived social status. In particular, there is evidence suggesting that, in China, White people are perceived as having a relatively higher social status^21,22^. The stronger oculomotor interference elicited by White faces in Chinese participants would then indicate a more robust attentional response to eye-gaze signals conveyed by faces belonging to individuals with a relatively higher social status.

Importantly, the lack of difference in oculomotor interference displayed by Italian participants in response to White and Asian faces is unlikely to reflect insensitivity to the ethnic membership. Indeed, previous data showed that ethnicity modulates social attention when the focus is on a White versus Black face comparison. Notably, this finding has been demonstrated both in studies using covert attention paradigms^18,22^ and in studies using the oculomotor interference paradigm^37^. The present results are in line with previous studies confirming that Western individuals exhibit a similar social attention behaviour to White and Asian faces^22,38^. This, in turn, may suggest that Asian and White individuals may be associated with a rather similar social status, at least in the Western countries where the few available studies have been carried out. Alternative predictions based on different factors such as low-level perceptual properties of the face stimuli (i.e., generalized stronger oculomotor interference for faces belonging to a specific ethnicity) or perceptual familiarity (i.e., stronger oculomotor interference for ingroup faces) would not be consistent with the observed pattern of findings. Indeed, as concerns low-level factors (differences in the perceptual features of the faces belonging to the two ethnic groups), these were also safely ruled out based on the consideration that, if present, these factors should predict symmetric effects. For instance, if White (or Asian) faces have some specific and unique perceptual features, then a stronger oculomotor interference for White (or Asian) faces should be expected irrespective of the respondents’ group membership. Our data are not consistent with this possibility since they evidence a stronger oculomotor interference for one face type but only in a specific group of respondents (i.e., we found an asymmetric effect). As concerns familiarity, it has been shown using a manual response task, that gaze cueing is stronger for familiar faces^11^. In the current experiment, we can assume that in-group faces should be more familiar to participants. Hence, if familiarity were involved, this should result, if anything, in a stronger oculomotor interference for Asian faces in Chinese participants. This was clearly not the case. Hence, the social meaning of ethnicity in a given cultural context is likely to be one key factor modulating the oculomotor interference effect in the present study.

Ethnicity is a powerful determinant of how members of societies perceive others. Indeed, individuals make relatively stable evaluations of the social status of one’s own and other ethnic groups^39^. Thus, when viewing a face belonging to a specific ethnicity, perceived social status is likely to be quickly activated^40^. Sensitivity to the social status of individuals seems to be a relevant trait in human nature that appears early during development^41^. As regards social attention, it has been shown that observers tend to be more sensitive to the gaze of faces belonging to individuals being higher in the social hierarchy^3,14,15^. Thus, one possibility is that perceived social status acted as a major determinant in shaping the present pattern of findings^21,22,42^. This interpretation, however, needs to be taken with caution in that perceived group status differences were not assessed in the current study and status is also a multifaceted notion with several antecedents (e.g., dominance, prestige) and correlates^40^. Future studies will possibly assess and/or manipulate social status perception to further clarify its possible role in shaping the oculomotor interference effect.

An emerging literature has addressed the role of cultural differences in visuo-spatial attentional mechanisms involved in face processing^43^. In this regard, previous studies have consistently shown that Western and Eastern individuals tend to explore faces in a different manner. More specifically, Western individuals are more focused on the mouth region, whereas Eastern individuals tend to look more over the eyes and central parts of the face^44,45^. Interestingly, however, a recent study^46^ employing a free-viewing paradigm with faces of different ethnicity reported no significant interactions of face ethnicity (Asian vs. White) with ethnicity of the participants (Japanese vs. British). This, in turn, does not favor the view according to which the present findings simply reflect basic differences in face scanning between Eastern and Western individuals. More in general, differences in overall performance in attentional tasks between Asian and Western participants have been documented in previous studies. In particular, Asian participants have been reported to respond more slowly than Western participants. This finding, however, has been interpreted as reflecting response biases rather than attentional differences driven by cultures^47,48^. In our data we observed a similar pattern, but this can hardly be interpreted as the driving factor underlying how individuals belonging to the two cultural groups differently respond to White and Asian faces. In sum, although cultural differences in visual exploration are robust and important as a general factor, the available evidence does not seem to support a specific role in influencing the modulation of gaze-driven attentional and oculomotor responses as a function of the ethnicity of face stimuli.

In conclusion, our study provides support for the view that social attention can be influenced by social variables and that major differences can arise depending on the socio-cultural context. The current results both corroborate and enrich previous evidence, confirming that the oculomotor interference paradigm employed here is a well-suited instrument to reveal social modulations of social attention behaviour.

## Methods

### Participants

Thirty Chinese participants (*Mean age* = 22.13 years, *SD* = 3.43, 22 females) from Guangzhou University and thirty White Italian participants (*Mean age* = 19.33 years, *SD* = 1.49, 22 females) from the University of Padova took part in the experiments. All participants were naïve to the purpose of the experiments. Moreover, they had normal or corrected-to-normal vision. In particular, as for Chinese participants, twenty of them completed the experiment wearing glasses and other four wearing contact lenses while, as for Italian participants, eight of them completed the experiment wearing glasses and other three wearing contact lenses. All participants provided a written, signed informed consent. All methods were approved by the “Ethics Committee for Psychological Research” at the University of Padova and by the “Institutional Review Board of the Educational School” at Guangzhou University. All methods were performed in accordance with the relevant guidelines and regulations and with the Declaration of Helsinki.

### Apparatus and stimuli

Because there is evidence that changes in the experimental setting can differentially affect eye-tracking data as a function of the ethnicity of the participants^49^, the same person (i.e., the first author of this paper) supervised data collection in both countries, thus ensuring consistency across experiments. Moreover, both apparatus and stimuli were identical for both Chinese and Italian participants. Specifically, eye movements were recorded monocularly at 1000 Hz with an EyeLink 1000 Plus (SR Research Ltd. Ottawa, Canada) within a room illuminated with neon ceiling lights. Stimuli were presented on a 24-inch monitor with a resolution of 1280 × 1024 pixels and a refresh rate of 120 Hz. Participants sat 70 cm from the monitor and their heads were stabled with a chinrest. The experiment was programmed and run in Experiment Builder (SR Research Ltd. Ottawa, Canada). Before the beginning of each experimental session, a five-point (HV5) calibration/validation procedure was completed. The validation procedure was accepted only when the worst point error was smaller than 1.5° and the average error smaller than 1°. The mean worst point and the mean average error in Chinese participants were 0.72° (*SD* = 0.28) and 0.44° (*SD* = 0.23), respectively, while, for Italian participants, they were 0.71° (*SD* = 0.30) and 0.40 (*SD* = 0.19), respectively. Two-tailed independent *t* test analyses showed that neither the mean worst point error, *t*(58) = 0.124, *p* = 0.902, *d* = 0.032, nor the mean average error, *t*(58) = 0.819, *p* = 0.416, *d* = 0.211, differed between the two samples, thus indicating that the calibration/validation procedure led to similar outputs in both experiments.

Face stimuli consisted of sixteen 3D full-coloured faces created with FaceGen 3.1 software (4 White females, 4 White males, 4 Asian females, 4 Asian males). For each face, there were three different versions: one with direct gaze, one with left-averted gaze, and one with right-averted gaze. Each face subtended about 14.4° in width and 16.8° in height and was presented over a grey background (RGB = 180, 180, 180).

### Design and procedure

Both design and procedure were identical for both Chinese and Italian participants. Data collection was performed by a person of the same ethnicity as the participants. At the beginning of each trial, participants were required to fixate on a black central dot (0.45° in diameter) for a drift checking procedure, aimed to ensure that they fixated the centre of the screen. This consisted in the experimenter pressing the spacebar when the participant’s fixation was exactly on the central dot. Then, a trial started with a central black fixation dot (0.45° in diameter) flanked by two black placeholders (0.85° of side). The two placeholders were placed 10° leftwards and rightwards, respectively, from the central fixation dot (i.e., the centre of the screen), and at the same height as the fixation dot. Then, a directed-gaze face appeared at the centre of the screen, with the two eyes vertically aligned with the fixation dot. After 1000 ms, the directed-gaze face was replaced by the same face with the gaze averted either leftwards or rightwards. After either a SOA of 0 ms (i.e., simultaneously) or 900 ms, the fixation dot changed to either a “+” or a “×” symbol (i.e., the instruction cue; 0.45° height × 0.45° width), namely the same symbol rotated, or not, by 45°. Participants were instructed to make a saccade towards either the left or the right placeholder and to ignore the gaze direction of the face since it was task-irrelevant. The saccadic eye movement had to be executed as soon as the instruction cue appeared. Half of the participants were instructed to perform a leftwards saccade in response to the “+” symbol and a rightwards saccade in response to the “×” symbol. For the other half of the participants, the directional instructions were reversed. Participants were provided with 1000 ms to perform the requested saccade. Finally, a blank screen appeared for 1500 ms before the next drift checking procedure (see Fig. 1). Participants were instructed to execute fast and directionally accurate (i.e., left or right) saccades, and to ignore the task-irrelevant central faces. All participants completed a practice block of 10 trials, followed by two experimental blocks of 128 trials each (i.e., 256 experimental trials in total). Participants were allowed a short break in between the blocks, although they were required to keep their head placed on the chinrest. Within each experimental block there was an equal number of trials resulting from the combination of the different experimental factors. The whole experimental session lasted about 45 min.

### Data handling

Data handling was identical for both Chinese and Italian participants. Saccades were defined as eye movements exceeding 30°/s in velocity and 8000°/s in acceleration and with a minimum amplitude of 2°. On each trial, the first saccade detected after the onset of the instruction cue was extracted. Then, only the saccades which did not contain a blink were analysed, to avoid any potential impact of blinks on the subsequent analyses of saccadic parameters. In particular, two types of blinks have been identified, namely blinks performed during a saccadic eye movement and blinks executed prior to a saccadic eye movement. Trials including either type of blinks have been excluded from the analyses. On average, the mean percentage of blinks made within the extracted saccades was 4.62% (*SD* = 5.68) in the Chinese sample, and 1.30% (*SD* = 3.12) in the Italian sample. Despite these low percentages, in order to confirm that blinks were not associated with specific experimental conditions, exploratory 2 × 2 × 2 repeated-measures ANOVAs with congruency (spatially congruent vs. incongruent), ethnicity (Asian vs. White), and SOA (0 vs. 900 ms) as within-participant factors were conducted on the mean percentage of blinks in both samples. In Chinese participants, all results were not significant, (*F*s < 3.301, *p*s > 0. 080), including the theoretically-relevant congruency × ethnicity interaction (*F* = 1.465, *p* = 0.236), and the three-way interaction (*F* < 1, *p* = 0 .572). The same pattern emerged also in Italian participants, since all results were not significant, (*F*s < 2.724, *p*s >  0.110), including the theoretically-relevant congruency × ethnicity interaction (*F* < 1, *p* =  0.684), and the three-way interaction (*F* < 1, *p* = 0.549). In addition, a further exploratory ANOVA was performed with the between-participants group factor (Chinese vs. Italian), confirming that none of the interactions including congruency and ethnicity were significant (*F*s < 1.629, *p*s > 0.207), as well as the four-way interaction (*F* < 1, *p* = 0.755). The only significant result involving the group factor was its main effect, *F*(1, 58) = 7.953, *p* = 0.007,* η*^*2*^_*p*_ = 0.121, indicating that blinks were overall more frequent among Chinese than Italian participants. As for the blinks executed prior to a saccadic eye movement, this kind of trials—in which a blink occurred after the onset of the instruction cue and prior to a correct saccade, was very rare in both Chinese (0.45% of total trials) and Italian participants (0.22% of total trials), and importantly, were not significantly different across the two groups (*t*(58) = 1.45, *p* = 0.15, *d* = .374). Saccades made towards the opposite spatial location as that indicated by the instruction cue (i.e., saccadic directional errors) were analysed separately (8.27% of trials of the sample of Chinese participants; 6.19% of trials in the sample of Italian participants). Correct saccades with a latency falling outside the 80–1000 ms range, were discarded from the analyses (0.54% of trials in the sample of Chinese participants; 0.04% of trials in the sample of Italian participants). Trials in which the instruction cue and the averted gaze of the facial stimulus conveyed the same spatial locations (i.e., right–right or left–left) were classified as congruent trials, the others as incongruent trials. Trials with missing data (i.e., trials in which no saccades were detected) were very rare in both Chinese (1.08% of total trials) and Italian participants (0.07% of total trials) and were not analyzed further.

## Data Availability

The datasets generated during this study are available on OSF at https://doi.org/10.17605/OSF.IO/CB65X.
